# Clinicohematological Spectrum of Chronic Myeloid Leukemia: A Cross-Sectional Study From a Tertiary Care Hospital in North India

**DOI:** 10.7759/cureus.86680

**Published:** 2025-06-24

**Authors:** Sarah Arnestina, Rupinder Kaur, Joseph M John

**Affiliations:** 1 Department of Pathology, Maharishi Markandeshwar Medical College and Hospital, Solan, IND; 2 Department of Pathology, Christian Medical College and Hospital, Ludhiana, IND; 3 Department of Pathology, Maharishi Markandeshwar Institute of Medical Science and Research, Ambala, IND; 4 Department of Hematology and Oncology, Christian Medical College and Hospital, Ludhiana, IND

**Keywords:** bcr-abl-1, bone marrow aspiration and biopsy, chronic myeloid leukemia, cml, mpn, myeloproliferative neoplasms, peripheral blood film

## Abstract

Introduction: Myeloproliferative neoplasms (MPNs) are a group of clonal hematopoietic stem cell disorders characterized by proliferation of one or more myeloid lineages in the bone marrow, resulting in an increased number of mature and immature cells in the peripheral blood and bone marrow. Chronic myeloid leukaemia (CML) is one of the commonest clonal myeloproliferative neoplasms encountered in clinical practice. It is characterized by a reciprocal rearrangement and fusion of the BCR genes on chromosome 22 and the ABL gene on chromosome 9.

Materials and methods: A cross-sectional study on the clinicohematological profile of chronic myeloid leukemia patients was conducted over five years in the hematology section of the pathology department in a tertiary care centre in North India. Detailed clinical history and examination, laboratory investigations of all the patients, along with molecular and cytogenetic studies, were noted from the patients' file records and bone marrow request forms. Peripheral blood film (PBF), bone marrow aspiration (BMA), clot section, and bone marrow biopsy slides were studied in detail. Frequencies and proportions were calculated based on all the above parameters, with relevant statistical analysis and final diagnosis were made and classified according to the WHO 2016 classification.

Results: There were a total of 76 patients with CML, out of which 52 (69%) were in the chronic phase, 6 (8%) in the accelerated phase, and 18 (24%) in blast crisis. Leucocytosis with myeloid predominance was seen both on peripheral blood film as well as in bone marrow aspiration and biopsy slides in all of the cases. Cytogenetic studies showed BCR-ABL1 positivity in the majority of the patients (82.8%; n=63).

Conclusion: Most of the data available on MPNs is from studies in developed countries. There is only limited literature in India due to a lack of a centralized registry. The present study was an attempt to categorize CML as a group based on symptoms, morphology, and cytogenetics at a tertiary care hospital in a real-world setting.

## Introduction

Myeloproliferative neoplasms (MPNs) encompass a group of clonal disarrays, resulting from aberrant stem cell myeloproliferation and are characterized by granulocytosis, erythrocytosis with or without thrombocytosis [1]. BCR-ABL positive chronic myelogenous leukemia (CML) is the commonest entity encountered in clinical practice in the Asian subcontinent and is usually associated with the BCR-ABL1 fusion gene present on the Philadelphia chromosome t(9;22) encoding for proteins which have persistently enhanced tyrosine kinase activity [2]. CML is primarily a neoplasm of adults, with peak incidence occurring between the fifth and seventh decades of life; however, a few subtypes are seen in childhood as well [3]. 

Annual incidence studies in India on CML reported an age-adjusted rate ranging from 0.71-2.2/100,000 population in males and 0.53-1.6/100,000 population in females [4-5]. The majority of the patients developed splenomegaly or hepatomegaly due to sequestration of excess blood cells or proliferation of abnormal hematopoietic cells [6]. This study was undertaken since published epidemiological data regarding incidence rates for CML are scarce for the Indian population, as there is no well-defined cancer registry for these neoplasms, and molecular diagnosis is not available to many clinicians.

## Materials and methods

This was a cross-sectional study done in the Hematology section of the Department of Pathology over a period of five years (including both retrospective and prospective periods) in Christian Medical College and Hospital, Ludhiana. A total of 150 diagnosed patients of MPNs were evaluated, of which 76 cases of CML were included in this study. 

All suspected and diagnosed patients of MPNs were included. If bone marrow slides were not available for retrospective cases, those cases were excluded. Relevant laboratory investigations like complete blood count (CBC), liver function tests (LFT), and renal function tests (RFT) were noted down from the bone marrow request records as well as from the patients' records file. Peripheral blood film (PBF), bone marrow aspirate (BMA) smears, and trephine biopsies were studied in detail for all the cases. Smears were reviewed under 100X, 400X, and 1000X. A 500-cell count was done on BMA smears, and an average was taken. The details for both PBF and BMA smears were documented as per protocol. Special stains such as periodic acid-Schiff (PAS) and myeloperoxidase (MPO) stains were used wherever required. Cytogenetics and mutation studies involving gene mutation and chromosomal rearrangement were also taken into account wherever available.

## Results

Out of 76 cases, 52 cases were CML in the chronic phase (69%), followed by 18 cases of CML in the blast phase (24%) and six cases of CML in the accelerated phase (8%). The majority of the patients in our study were males with a M: F ratio of 4.8:1. The patients' age ranged from 12-88 years, with the mean age of 49.9 ± 16.1 years.

The most common clinical symptom seen in patients with CML in all the phases was weakness (26%), closely followed by fever (25%) and abdominal pain. Other clinical features seen were loss of appetite, cough, and abdominal mass. Splenomegaly was the most consistent finding in 41 patients (78.8%), showing a maximum spleen span of 35 cm under the left costal margin. A total of 25 patients had hepatomegaly (48%), with the maximum span of the liver of 17.5 cm under the right costal margin. Five patients had pallor, two showed axillary and cervical lymphadenopathy, and one had ecchymosis.

A renal function test and liver function tests were done for all the patients, and were within normal limits. Lactate dehydrogenase (LDH) levels were elevated in 39 patients in CML in the chronic phase, ranging from 222-4344 U/L (mean 1392 U/L), and in 14 patients with CML with blast crisis, ranging from 290-4948U/L, with a mean LDH of 1474 U/L.

The CBC and Bone marrow findings of these patients are summarized in Table 1.

**Table 1:** CBC and bone marrow findings in patients with CML CBC: complete blood count; CML: chronic myeloid leukaemia; CP: chronic phase; AP: accelerated phase; BP: blast phase; TLC: total leukocyte count; PBF: peripheral blood film; BMA: bone marrow aspiration

Parameter	CML in CP (N=52)	CML in AP (N=6)	CML in BP (N=18)
Hemoglobin (g/dL)	10.7 ± 2.2	8.4 ± 2.2	8.1 ± 3.0
TLC (10³/µL)	131 ± 116	177 ± 174	92 ± 123
Platelets (10³/µL)	383 ± 255	331±1239	675 ± 607
PBF blast count (%)	2 (0-8)	8 (1-15)	32 (14-86)
BMA blast count (%)	2 (0-7)	10 (5-14)	58 (21-92)
BCR-ABL testing			
BCR-ABL-positive	44	5	14
Unsatisfactory	2	1	0

Out of 52 bone marrow aspirates in cases in chronic phase, 42 cases (81%) were solidly cellular to hypercellular for age. Two cases yielded scanty fragments while eight cases (15%) showed dry tap. A total of 42 cases showed myeloid predominance (M:E 1.3:1 to 99:1) with increased megakaryopoiesis showing prominence of dwarf forms. Additionally, 18 cases showed eosinophilia as well.

The average measurement of the trephine biopsy was 2.8 cm in length. The trephine biopsies showed similar results to the corresponding aspirates. The reticulin stain showed 25% of cases showing diffuse fibrosis (3+), followed by 17% of cases showing mild fibrosis (1+), and 12% of patients showing 2-3+ fibrosis. Collagen fibrosis was demonstrated in one case by Masson’s Trichrome stain. 

**Figure 1:**
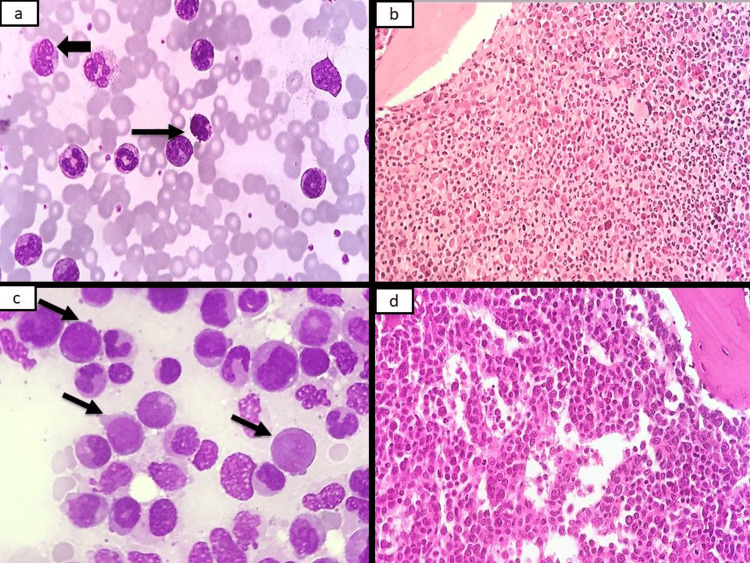
PBF and bone marrow findings in CML a. Photomicrograph showing basophil (arrowhead) and eosinophil (arrows) along with other myeloid precursors (Leishman 1000x). b. Photomicrograph showing solidly cellular marrow with an increase in myeloid precursors and prominence of eosinophils (H&E 400x). c. Bone marrow aspirate showing presence of blasts (arrows) in a case of CML in the accelerated phase (MGG 1000x). d. Trephine biopsy showing sheets of monocytoid cells with vesicular nuclei, prominent nuclei and abundant cytoplasm in a case of CML in monocytoid blast crisis (H&E 400x). PBF: peripheral blood film; CML: chronic myeloid leukemia

In the accelerated phase, WBCs showed marked leucocytosis with mainly myeloid predominance along with basophilia, neutrophilia, and eosinophilia. The blasts in the PBF were 12-14 microns in size with a high nuclear-to-cytoplasmic (N/C) ratio, immature chromatin, 1-2 prominent nucleoli, and scant cytoplasm. No Auer rods were seen. The marrows were solidly cellular to hypercellular in all cases, showing marked myeloid predominance with an increase in blast count ranging from 5-14% with myeloid predominance (M:E, 2.4:1 to 48:1). The average measurement of the trephine biopsy was 3.3 cm in length. The biopsies in all the cases showed similar findings, with 50% of cases showing diffuse fibrosis (3+) on reticulin stain (Figure 1).

In blast crisis, PBF showed hyperleucocytosis in 12 cases with marked myeloid predominance. Three cases had normal TLC while 3 cases had leucopenia. Out of the 18 cases, seven cases had aparticulate smears. The cellularity was hypercellular to solidly cellular in 11 cases. Six cases were in lymphoid blast crisis (33%) and four cases were in myeloid blast crisis (22%), out of which two showed monocytoid differentiation; the remaining eight cases were negative for both MPO and PAS staining. The average measurement of the trephine biopsy was 3.2 cm in length, and all were cellular for age. More than 50% of cases showed diffuse fibrosis (3+).

Cytogenetic studies showed BCR-ABL1 positivity in the majority of the patients (82.8%; n=63). The remainder of the patients did not get tested due to financial constraints.

## Discussion

Studies from India have reported sparse literature on epidemiology and presentation of haematological malignancies [7-8]. There were a total of 76 cases of CML in the present study, of which the majority were in chronic phase, which was similar to studies done by Ahmed et al. [9], Anand et al. [10], Malhotra et al. [11], and Benchikh et al [12]. In our study, cases of CML in accelerated phase were the least common, comparable to other studies [9-11].

The percentage of cases of blast crisis was higher compared to the accelerated phase in our study, which could be attributed to late presentation of patients or late referral to a tertiary care hospital.

The age for presentation for the accelerated phase in our study was comparable to a few Indian studies, such as Irfan et al. [13], Ray et al. [14], Malhotra et al. [11], and Kantarjian et al. [15], while the data from Western countries showed late presentation of the disease as shown in studies by O’Brien et al [16] and Hochhaus et al [17]. There was a male preponderance in our study, which was also seen in all the other studies. 

Splenomegaly was present in 78% of the patients, and 48% of the patients had hepatomegaly in the chronic phase, which was slightly lower in comparison to Malhotra et al. [11], who reported a higher prevalence (89%) of splenomegaly and hepatomegaly (61%) in their study. However, Kantarjian et al. [15] reported splenomegaly in only 46% of patients, which was lower than in our study.

In the present study, 46 cases (88%) in the chronic phase had leucocytosis showing left shift with basophilia and eosinophilia, which was similar to findings by Kantarjian et al [15] and Ahmed et al [18].

In the CML in AP, all the cases had leucocytosis with left shift. The median blast count in the peripheral blood was 6%, which was comparable to the study done by Kantarjian et al (2%) [15]. Ahmed et al. [18] in their study on seven cases of accelerated phase found blast count ranging from 12-16%. In CML in BP, the median age was 41.5 years in our study, whereas it was a decade older in the study done by Kantarjian et al [15].

The most common symptom in patients of CML in blast crisis in our study was weakness (29%), followed by fever (23%). In contrast to this, the study done by Malhotra et al. [11] reported fever in 67%, along with lymphadenopathy in 53% as opposed to the present study. A total of 14 patients had splenomegaly (78%) and 10 had hepatomegaly (56%) in our study.

In the present series, the blasts ranged from 0-86%% with the median blast count of 33.5%, which was in concordance with the study done by Kantarjian et al [15]. In the aspirate, the blasts ranged from 21-92%, with the mean blast count of 58%. Ahmed et al. [18] in their case study, reported blast count to range 32-97% with a mean blast count of 64±33%.

In the present series, a BCR ABL gene study was done in 66 cases of CML, out of which 63 cases were positive (95.4%), whereas Anand et al. [19] from India reported 100% positivity of the BCR ABL gene.

## Conclusions

To our knowledge, much of the data available on MPNs is from studies conducted in Western countries. There is only limited literature in India due to a lack of a centralized registry. The present study was an attempt to categorise CML as a group based on symptoms, morphology, and cytogenetics at a tertiary care hospital in a real-world setting. 
